# Prognostic Value of Elevated Copeptin and High-Sensitivity Cardiac Troponin T in Patients with and without Acute Coronary Syndrome: The ConTrACS Study

**DOI:** 10.3390/jcm9113627

**Published:** 2020-11-11

**Authors:** Hanna Waldsperger, Moritz Biener, Kiril M. Stoyanov, Mehrshad Vafaie, Hugo A. Katus, Evangelos Giannitsis, Matthias Mueller-Hennessen

**Affiliations:** 1 Department of Internal Medicine III, Heidelberg University Hospital, 69120 Heidelberg, Germany; hanna.waldsperger@t-online.de (H.W.); moritz.biener@med.uni-heidelberg.de (M.B.); kiril.stoyanov@med.uni-heidelberg.de (K.M.S.); mehrshad.vafaie@med.uni-heidelberg.de (M.V.); hugo.katus@med.uni-heidelberg.de (H.A.K.); Evangelos_Giannitsis@med.uni-heidelberg.de (E.G.); 2 DZHK (German Centre for Cardiovascular Research), partner site Heidelberg/Mannheim, 69120 Heidelberg, Germany

**Keywords:** copeptin, cardiac troponin, high sensitivity, acute coronary syndrome, emergency department, prognosis, risk stratification

## Abstract

Aims: We aimed to assess the prognostic role of copeptin in patients presenting to the emergency department with acute symptoms and increased high-sensitivity cardiac troponin T. Methods: A total of 3890 patients presenting with acute symptoms to the emergency department of Heidelberg University Hospital were assessed for increased hs-cTnT (>14 ng/L) from three cohorts: the Heidelberg Acute Coronary Syndrome (ACS) Registry (*n* = 2477), the BIOPS Registry (*n* = 320), and the ACS OMICS Registry (*n* = 1093). In a pooled analysis, 1956 patients remained, comprising of 1600 patients with ACS and 356 patients with non-ACS. Results: Median follow-up was 1468 days in the ACS cohort and 709 days in the non-ACS cohort. Elevated copeptin levels (>10 pmol/L) were found in 1174 patients (60.0%) in the entire cohort (58.1% in ACS and 68.5% in non-ACS, respectively) and mortality rates were significantly higher than in patients with normal copeptin levels (29.0% vs. 10.7%, *p* < 0.001). In a multivariate Cox regression, elevated copeptin was independently associated with all-cause death in the ACS (HR = 1.7, 1.3–2.3, *p* = 0.002) and non-ACS cohort (HR = 2.7, 1.4–5.0, *p* = 0.0018). Conclusion: Copeptin may aid in identifying patients at risk for adverse outcomes in patients with increased levels of hs-cTnT in ACS patients and in non-ACS conditions.

## 1. Introduction

Copeptin is a marker of endogenous stress and the most promising application in clinical practice is the combination with cardiac troponin (cTn) for early rule-out of acute myocardial infarction (AMI) in patients presenting with symptoms suggestive of acute coronary syndrome (ACS) [1,2,3]. Accordingly, a dual-marker strategy (DMS) with normal levels of copeptin and conventional cTn is recommended by guidelines for non-ST-elevation myocardial infarction [4,5]. In addition, the DMS has been found to allow a reliable and safe discharge of patients using high-sensitivity (hs) cTn in several studies, including randomized interventional trials and large multicenter registries [5,6,7,8]. With the potential increase of copeptin testing for rule-out of AMI in emergency departments (EDs), there is also an emerging need to interpret biomarker findings that do not qualify for immediate rule-out, e.g., elevated copeptin or hs-cTnT. Given the paucity of existing evidence the interpretation of these non-diagnostic constellations remains unclear and may cause improper conclusions among physicians [9]. In contrast to cTn, the release of copeptin is unspecific and may be stimulated by numerous acute clinical pathologies, including acute heart failure, pulmonary embolism, and sepsis [10,11,12,13]. Previously, elevated copeptin values in patients with suspected ACS but also non-ACS conditions were found to confer incremental prognostic information beyond an increased hs-cTn [10,14]. However, Stallone et al. found that the association of elevated copeptin levels with mortality may be largel, y explained by age and comorbidities [9]. Thus, the aim of the ConTrACS study was to assess the prognostic impact of copeptin in patients with increased hs-cTnT presenting to the emergency department with acute symptoms, including ACS and non-ACS conditions.

## 2. Methods

### 2.1. Study Population

The study cohort consisted of symptomatic patients with increased hs-cTnT (>14 ng/L) presenting with ACS or non-ACS conditions to the ED of Heidelberg University Hospital. We screened three existing cohorts for study eligibility: 2477 patients from the Heidelberg ACS Registry presenting from June 2008 to November 2011 to the ED with a working diagnosis of ACS, 320 patients from the BIOPS Registry presenting to the ED with new onset of dyspnea or worsening of chronic dyspnea (May 2013 to November 2014), and 1093 patients from the ACS OMICS Registry (August 2014 to July 2017) presenting with symptoms suggestive of AMI. In total, 1956 patients qualified for study inclusion. Detailed information on the initial study cohorts and a study flow-chart (Supplementary Materials Figure S1) can be found in the Supplementary Materials section.

The final diagnosis was adjudicated by an expert committee of two independent cardiologists, with discrepancies solved by discussion with a third cardiologist. Timing and selection of diagnostic or therapeutic procedures were left at the discretion of the attending physician. Adjudicated diagnosis of ACS required evidence of clinical ischemia with symptoms of chest pain or angina equivalent, such as dyspnea, and at least one of the following criteria: new ischemic ECG changes or new imaging evidence of wall motion abnormalities or detection of a coronary artery stenosis ≥70% on cardiac catheterization. In conjunction with evidence of clinical ischemia, the diagnosis of AMI required an increased cTn value above the 99th percentile cut-off together with a rise and/or fall of cTnT as suggested by the Fourth Universal Definition of MI [15]. In the absence of an atherothrombotic cause and evidence of a supply/demand imbalance, AMI patients were further classified as type 2 MI.

Follow-up was performed at least 30 days after discharge by telephone calls, in written form, by review of the hospital information system or by obtaining information of survival of the local residents’ registration offices. The study was carried out according to the principles of the Declaration of Helsinki. Each registry was approved by the local ethics committee of the University of Heidelberg (No. 3302003, S1172013, and S3512015) and written informed consent was obtained from all patients. 

### 2.2. Laboratory Analysis

Blood samples were collected in plasma tubes at presentation. Following centrifugation, samples were frozen at −80 °C for further analysis. Measurement as part of the clinical routine was performed in 1593 patients for hs-cTnT and 172 patients for copeptin, while remeasurement from frozen samples was performed in 363 patients for hs-cTnT and 1784 for copeptin. hs-cTnT was measured using the Elecsys^®^ Troponin T high sensitive assay (Roche Diagnostics, Mannheim, Germany) on a cobas e411 immunoassay analyzer. Limit of Blank, Limit 99th of Detection, 10% coefficient of variation (CV), and percentile cut-off values were determined to be 3, 5, 13, and 14 ng/L [16,17]. Measurement of copeptin was performed with the copeptin proAVP assay on the KRYPTOR compact plus (BRAHMS Thermo Fisher Scientific, Hennigsdorf, Germany). Detection limit, precision of 20% CV, and 95th cut-off values were found to be 0.69, 1.08, and 9.8 pmol/L for the copeptin proAVP assay [2,18].

### 2.3. Outcome Measures

In accordance to previously tested copeptin thresholds [2,7], a cut-off of 10 pmol/L was used for prognostic evaluation of copeptin. All-cause mortality was defined as the primary outcome, whereas the combined end-point of all-cause death, MI, and stroke were used as secondary outcomes. The Global Registry of Acute Coronary Events (GRACE) risk-score was calculated as previously described [19].

### 2.4. Statistical Methods 

Continuous variables are presented as median with interquartile range for a non-normal distribution or as means (±95% confidence intervals) for normally distributed data. For comparison of continuous parameters, the Mann–Whitney U-test was used, whereas a Chi-square test was applied for categorical parameters. For prognostic assessment, mortality and the combined end-point of all-cause death, MI, and stroke in short- and long-term follow-up was assessed using Cox regression and plotted in Kaplan–Meier survival plots. Receiver-operating-characteristic (ROC) curves were used to determine the prognostic performance with area-under-the-curves (AUCs) for prediction of adverse outcomes. All hypothesis testing was two-tailed and *p*-values <0.05 were considered statistically significant. Statistical analyses were performed using MedCalc 15.6 (MedCalc Software, Ostend, Belgium) and SPSS 22.0 (IBM, Armonk, NY, USA).

## 3. Results

### 3.1. Baseline Characteristics

The entire cohort of 1956 patients comprised of 1600 patients with ACS and 356 patients with non-ACS. Elevated copeptin levels (>10 pmol/L) on admission were found in 1174 patients (60.0%) in the entire cohort as well as 58.1% in ACS and 68.5% in non-ACS, respectively. Detailed clinical characteristics for the entire cohort and the ACS and non-ACS sub-cohorts can be found in Table 1. Patients with elevated copeptin were significantly older and more frequently presented with dyspnea, non-ACS conditions, and ST-elevation myocardial infarction (STEMI) diagnosis. Moreover, these patients had a more recent onset of symptoms (<3 h), higher creatinine levels, and a higher GRACE-risk score. An overview on categories of adjudicated non-ACS diagnoses is provided in Supplementary Materials Table S1.

### 3.2. Outcomes

Median follow-up was 1274 (IQR 433–2407) days in the entire cohort as well as 1468 (IQR 497–2542) days in the ACS and 709 (IQR 385–1420) days in the non-ACS cohort. Follow-up for the primary outcome was obtained in 100% after 30 days and 98.82% after 1 year.

Detailed numbers of the different outcomes according to normal or elevated copeptin levels in ACS and non-ACS patients can be found in Table 2. In the entire cohort, all-cause death was observed in 21.7%, with a significantly higher mortality in patients with elevated copeptin compared to normal copeptin levels (29.0% vs. 10.7%, *p* < 0.001). Mortality rates after 30 days were low for patients with elevated hs-cTnT and normal copeptin levels (0.5%), whereas elevated levels of both hs-cTnT and copeptin had an almost 10-time higher mortality in short-term follow-up (4.9%, *p* < 0.001). Significantly higher mortalities in short- and long-term follow-up could also be found for ACS patients and non-ACS patients (Table 2). The composite of death, MI, and stroke showed a similar pattern between patients with increased and normal copeptin values, in the entire cohort (40.9% vs. 20.6%, *p* < 0.001) as well as in ACS (40.9% vs. 21.6%, *p* < 0.001) and non-ACS patients (41.0% vs. 14.3%, *p* < 0.001). 

### 3.3. Survival Analysis

**Figure 1:**
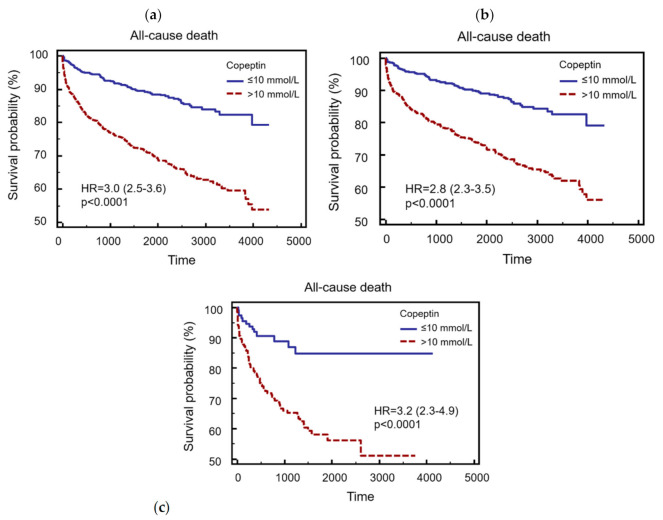
Kaplan–Meier curves for all-cause mortality in the entire cohort (**a**), in acute coronary syndrome (ACS) (**b**) and non-ACS patients (**c**).

Cox proportional hazard models for all-cause death revealed that elevated copeptin values had an independent prognostic value (HR = 2.0, CI 95% 1.5–2.5, *p* < 0.0001) after correction for age, eGFR, GRACE risk-score, recent symptom onset, symptom of dyspnea, hs-cTnT tertiles, and diagnosis of AMI in the entire cohort (Table 3A). Similarly, elevated copeptin was found to be an independent predictor for mortality in the ACS cohort (HR = 1.7, 95% CI 1.3–2.3, *p* = 0.0002) (Table 3B) and in the non-ACS cohort (HR = 2.7, 95% CI 1.4–5.0, *p* = 0.0018) (Table 3C). Figure 1 displays Kaplan–Meier curves for all-cause mortality in the entire cohort (A) as well as in ACS (B) and non-ACS patients (C). In addition, Cox proportional hazard models for the combined endpoint of death, myocardial infarction, and stroke can be found in Supplementary Materials Table S2. 

### 3.4. Sensitivity Analysis and Prognostic Performance

A copeptin cut-off >10 pmol/L exhibited a high negative predictive value of 89.3% and sensitivities of 80.2% for prediction of all-cause death in the entire cohort, while positive predictive values and specificities were lower (Supplementary Materials Table S3). The same pattern was found for adjudicated ACS and non-ACS diagnoses as well as for the combined endpoint of death, MI, stroke.

In ROC analysis, the combination of hs-cTnT with copeptin showed a superior prognostic performance in comparison to hs-cTnT alone for prediction of all-cause death in the entire (AUC = 0.68 vs. 0.56, *p* < 0.0001) as well as in the ACS cohort (AUC = 0.68 vs. 0.60, *p* = 0.0001) (Supplementary Materials Table S4A). Similar results were found for the combined endpoint of death/MI/stroke in the entire cohort and in the ACS cohort (Supplementary Materials Table S4A). In addition, the combination of the GRACE risk-score and copeptin showed a superior prognostic performance for prediction of all-cause death in comparison to the GRACE risk-score alone in the entire (AUC 0.77 vs. 0.76, *p* = 0.0087) and the ACS cohort (AUC 0.78 vs. 0.77, *p* = 0.04) (Supplementary Materials Table S4B). Contrarily, in the non-ACS cohort, no significant differences were found between the prognostic performance of hs-cTnT and the combination of hs-cTnT with copeptin as well as for GRACE score and the combination of GRACE score with copeptin (Supplementary Materials Table S4A,B).

## 4. Discussion

In the study, we evaluated the prognostic impact of copeptin in patients with increased hs-cTnT presenting with acute symptoms to the emergency department consisting of ACS and non-ACS conditions. We report several important findings:

First, the prevalence of elevated copeptin (>10 pmol/L) in patients with increased hs-cTnT (>14 ng/L) is high and may require special attention, particularly in the setting of more frequent copeptin testing for rule-out. Second, elevated copeptin was associated with higher age, creatinine levels, and GRACE risk score as well as STEMI diagnosis and recent onset of symptoms. Third, and most importantly, copeptin was independently associated with adverse outcomes after correction for relevant clinical parameters, such as renal function, age, GRACE risk score, hs-cTnT, symptom onset, and MI diagnosis, for all-cause death not only in ACS but also in non-ACS conditions.

The mechanism for copeptin release is not fully understood but may involve arterial underfilling, even in the absence of arterial hypotension as a possible mechanism. Accordingly, copeptin release has been reported in different cardiovascular diseases as well as in several acute non-cardiac conditions, including acute MI, coronary artery disease, pulmonary embolism, heart failure, sepsis, and stroke [10,13,20,21]. Regardless of the underlying pathology, an increase of copeptin was found to be associated with adverse outcomes and higher mortality rates [10,11,13]. However, sparse data are available on the independent prognostic information and on the optimal cutoff to predict complications.

Recently, Lattuca et al. found that copeptin on admission was an independent predictor of mortality in STEMI patients (unadjusted HR 3.1) with a superior prognostic value compared to peak cTnI (AUC of 0.74 vs. 0.60, *p* = 0.022 [11]. In support, Stengard et al. showed that copeptin and hs-cTnT were highly predictive of outcome in patients with suspected AMI [22]. However, concerning the ability to predict final infarct size determined by cardiac MRI, diverging results in AMI patients have been reported. While Ananth et al. showed that copeptin levels at admission independently predicted final infarct size [23], a sub-study of the DANAMI-3 trial found that there was no association of the copeptin/troponin ratio with the final infarct size [24].

Our findings on the prevalence of elevated copeptin are consistent with a previously reported prevalence of 22% in chest pain patients without an ACS [9]. While Stallone et al. found that elevated copeptin in these patients was accompanied by a three times higher mortality rate compared to normal copeptin levels, increased mortality risk could be largely explained by age and comorbidities. Thus, the authors concluded that no deviation in the treatment strategy in patients with elevated copeptin levels should be followed [9]. In contrast, we found that copeptin was independently related to adverse outcomes even in non-ACS conditions with an adjusted HR of 2.7. Similar findings were observed in the entire cohort and in the ACS cohort, with an adjusted HR of 2.0 and 1.7, respectively.

In recent years, copeptin has been predominantly evaluated as part of the DMS in combination with cTn [7] and seems to be beneficial for an accelerated rule-out of MI in patients with normal levels of less sensitive cTn and copeptin at presentation [4]. In addition, several studies recently showed the usefulness of the DMS with high-sensitivity cTn assays [6,7,8]. However, more frequent measurement of copeptin at presentation for rapid rule-out of MI in chest pain patients could eventually lead to a higher number of patients with copeptin values in the presence of elevated hs-cTnT at admission. Thus, appropriate interpretation of copeptin levels in the case of elevated hs-cTnT is important in the clinical setting in order to avoid improper conclusions of ED physicians. Especially, in patients presenting with acute symptoms to the ED, there is an essential need for early identification of patients who are at increased risk of adverse events and could benefit from accelerated treatment and intensified intrahospital monitoring or post-discharge follow-up. Conversely, early detection of patients at low risk even in the presence of elevated hs-cTnT values >14 ng/L is equally important to identify candidates for less aggressive medication and interventional treatment strategies, shortened monitoring, or early discharge options. Our study may thus aid ED physicians to optimize clinical management of these patients. While patients with elevated hs-cTnT and normal copeptin levels exhibited favorable outcomes in short- and long-term follow-up, rates of mortality and the combined endpoints were substantially higher in patients with elevated hs-cTnT and copeptin.

### Limitations

To evaluate the prognostic impact of copeptin in a clinical setting where copeptin may not be used diagnostically for rapid rule-out of AMI, the study cohort was restricted to patients with hs-cTnT above the 99th percentile cut-off. However, this leads to an inclusion bias with underrepresentation of patients with adjudicated non-ACS conditions and therefore, results on statistical significance in non-ACS patients may be hampered by the low number of patients in relation to ACS patients. In addition, the use of hs-cTnT as a covariate for risk stratification in our study may be biased by the fact that the study population already presents a higher risk population since the 99th percentile of hs-cTnT has been found be a strong discriminator for adverse outcomes.

## 5. Conclusions

Measurement of copeptin might aid in risk stratification for patients presenting with acute symptoms to the emergency department, irrespective of adjudicated ACS or ACS diagnoses. Even in the presence of elevated high-sensitivity cTn, copeptin provided an independent benefit to identify patients at increased risk in short- and long-term follow-up. In addition, use of copeptin for prognostication in the setting of increased cTn might complement the clinical use of copeptin for rule-out of AMI in the presence of normal cTn levels.

## Figures and Tables

**Table 1:** Baseline characteristics.

All Patients	All Patients (*n* = 1956)	Copeptin ≤10 pmol/L (*n* = 782)	Copeptin >10 pmol/L (*n* = 1174)	*p*-Value *
Age (years)	71 (61–79)	69 (58–77)	73 (63–79)	<0.0001
Male gender	1395 (71.3)	552 (70.6)	843 (71.8)	NS
Systolic pressure (mmHg)	147 (130–163)	150 (136–166)	145 (128–160)	<0.0001
Heart rate	76 (66–88)	75 (65–85)	78 (66–90)	0.0012
**Symptoms**				
Chest pain	1662 (85.0)	697 (89.1)	965 (82.2)	NS
Dyspnea	931 (47.6)	327 (41.8)	604 (51.4)	0.0025
Leading symptom AP	1350 (69.0)	564 (72.1)	786 (67.0)	NS
Leading symptom Dyspnea	307 (15.7)	79 (10.1)	228 (19.4)	<0.0001
**Onset of symptoms**				
<3 h	325 (16.6)	81 (10.4)	244 (20.8)	<0.0001
3–6 h	274 (14.0)	85 (10.9)	189 (16.1)	0.0025
>6–24 h	465 (23.8)	210 (26.9)	255 (21.7)	0.0225
>24 h	892 (45.6)	406 (51.9)	486 (41.4)	0.0007
**GRACE-Score**				
GRACE Score	119 (98–137)	110 (90–127)	126 (106–143)	<0.0001
GRACE-Score < 110	748 (38.2)	393 (50.3)	355 (30.2)	<0.0001
GRACE-Score 110–139	789 (40.3)	310 (39.6)	479 (40.8)	NS
GRACE-Score ≥ 140	419 (21.4)	79 (10.1)	340 (29.0)	<0.0001
**Laboratory**				
hs-cTnT 0 h (ng/L)	58.0 (26.0–229.0)	56.0 (21.0–251.0)	59.0 (28.0–206.9)	0.0422
hs-cTnT maximum (ng/L)	109.7 (34.0–501.0)	87.6 (27.0–442.0)	125.1 (41.0–577.0)	<0.0001
Copeptin (pmol/L)	13.7 (6.4–40.0)	5.4 (4.5–7.5)	30.7 (16.3–70.8)	<0.0001
Creatinine (mg/dL)	0.98 (0.81–1.25)	0.88 (0.76–1.03)	1.10 (0.87–1.46)	<0.0001
eGFR CKD-EPI (mL/min/1.73 m^2^)	73 (51–91)	84 (67–95)	63 (42–86)	<0.0001
eGFR CKD-EPI < 60 mL/min/1.73 m^2^	667 (34.1)	125 (16.0)	542 (46.2)	<0.0001
**Diagnosis**:				
ACS	1600 (81.8)	670 (85.7)	930 (79.2)	NS
non-ACS	356 (18.2)	112 (14.3)	244 (20.8)	0.001
AMI	1273 (65.1)	489 (62.5)	784 (66.8)	NS
Type I	1142 (89.7)	439 (89.8)	703 (89.7)	NS
Type II	121 (9.5)	45 (9.2)	76 (9.7)	NS
Type unclear	10 (0.8)	5 (1.0)	5 (0.6)	NS
non-AMI	683 (34.9)	293 (37.5)	390 (33.2)	NS
STEMI	339 (17.3)	83 (10.6)	256 (21.8)	<0.0001
NSTEMI	935 (47.8)	406 (51.9)	529 (45.1)	00316
Unstable angina	326 (16.7)	181 (23.1)	145 (12.4)	<0.0001
Cardiac non-coronary	272 (13.9)	84 (10.7)	188 (16.0)	0.0022
Non-cardiac	84 (4.3)	28 (3.6)	56 (4.8)	NS
**ACS**	**All patients** **(*n* = 1600)**	**Copeptin** **≤10 pmol/L** **(*n* = 670)**	**Copeptin** **>10 pmol/L** **(*n* = 930)**	*p*-Value *
Age (years)	71 (60–78)	69 (58–77)	72 (61–79)	<0.0001
Male gender	1166 (72.9)	483 (72.1)	683 (73.4)	NS
Systolic pressure (mmHg)	148 (132–163)	150 (137–166)	146 (128–160)	<0.0001
Heart rate	75 (66–86)	75 (65–84)	76 (66–88)	0.0217
**Symptoms**				
Chest pain	1497 (92.4)	629 (93.9)	850 (91.4)	NS
Dyspnea	658 (41.4)	256 (38.2)	402 (43.2)	NS
Leading symptom AP	1286 (80.4)	538 (80.3)	748 (80.4)	NS
Leading symptom Dyspnea	118 (7.4)	37 (5.5)	81 (8.7)	0.0206
**Onset of symptoms**				
<3 h	294 (18.4)	74 (11.0)	220 (23.7)	<0.0001
3–6 h	244 (15.3)	74 (11.0)	170 (18.3)	0.0003
>6–24 h	400 (25.0)	188 (28.1)	212 (22.8)	0.0377
>24 h	662 (41.4)	334 (49.9)	328 (35.3)	<0.0001
**GRACE-Score**				
GRACE Score	118 (96–136)	109 (90–126)	123 (104–141)	<0.0001
GRACE-Score < 110	650 (40.6)	343 (51.2)	307 (33.0)	<0.0001
GRACE-Score 110–139	634 (39.6)	263 (39.3)	371 (39.9)	NS
GRACE-Score ≥ 140	316 (19.8)	64 (9.6)	252 (27.1)	<0.0001
**Laboratory**				
hs-cTnT 0 h (ng/L)	79.9 (28.0–304.9)	74.0 (25.0–307.0)	87.0 (31.0–304.0)	0.0401
hs-cTnT maximum (ng/L)	166.9 (46.1–689.0)	118.5 (32.0–523.0)	212.8 (62.0–815.0)	<0.0001
Copeptin (pmol/L)	12.7 (6.1–37.4)	5.2 (4.5–7.3)	30.1 (15.8–74.1)	<0.0001
Creatinine (mg/dL)	0.96 (0.80–1.21)	0.88 (0.75–1.02)	1.05 (0.84–1.39)	<0.0001
eGFR CKD-EPI (mL/min/1.73 m^2^)	76 (55–92)	85 (68–96)	67 (44–88)	<0.0001
eGFR CKD-EPI < 60 mL/min/1.73 m^2^	485 (30.3)	98 (14.6)	387 (41.6)	<0.0001
**Diagnosis:**				
AMI	1273 (79.6)	489 (73.0)	784 (84.3)	0.0123
non-AMI	327 (20.4)	181 (27.0)	146 (15.7)	<0.0001
STEMI	339 (21.2)	83 (12.4)	256 (27.5)	<0.0001
NSTEMI	935 (58.4)	406 (60.6)	529 (56.9)	NS
Unstable angina	326 (20.4)	181 (27.0)	145 (15.6)	<0.0001
**Non-ACS**	**All patients** **(*n* = 356)**	**Copeptin** **≤10 pmol/L** **(*n* = 112)**	**Copeptin** **>10 pmol/L** **(*n* = 244)**	*p*-Value *
Age (years)	74 (65–80)	71 (60–78)	75 (67–81)	0.0029
Male gender	229 (64.3)	69 (61.6)	160 (65.6)	NS
Systolic pressure (mmHg)	145 (128–162)	147 (130–164)	143 (126–160)	NS
Heart rate	80 (67–95)	77 (65–91)	81 (68–99)	NS
**Symptoms**				
Chest pain	183 (51.4)	68 (60.7)	115 (47.1)	NS
Dyspnea	273 (76.7)	71 (63.4)	202 (82.8)	NS
Leading symptom AP	64 (18.0)	26 (23.2)	38 (15.6)	NS
Leading symptom Dyspnea	189 (53.1)	42 (37.5)	147 (60.2)	0.0062
**Onset of symptoms**				
<3 h	31 (8.7)	7 (6.2)	24 (9.8)	NS
3–6 h	30 (8.4)	11 (9.8)	19 (7.8)	NS
>6–24 h	65 (18.3)	22 (19.6)	43 (17.6)	NS
>24 h	230 (64.6)	72 (64.3)	158 (64.8)	NS
**GRACE-Score**				
GRACE Score	128 (107–143)	115 (93–132)	134 (116–146)	<0.0001
GRACE-Score < 110	98 (27.5)	50 (44.6)	48 (19.7)	<0.0001
GRACE-Score 110–139	155 (43.5)	47 (42.0)	108 (44.3)	NS
GRACE-Score ≥ 140	103 (28.9)	15 (13.4)	88 (36.1)	0.0002
**Laboratory**				
hs-cTnT 0 h (ng/L)	31.0 (21.0–61.0)	23.0 (16.3–47.8)	36.0 (24.0–65.0)	<0.0001
hs-cTnT maximum (ng/L)	34.0 (22.0–75.0)	25.0 (18.5–65.0)	40.0 (24.0–76.5)	0.0004
Copeptin (pmol/L)	18.7 (8.6–48.1)	6.0 (4.5–8.2)	33.1 (18.1–59.4)	<0.0001
Creatinine (mg/dL)	1.11 (0.87–1.46)	0.88 (0.76–1.07)	1.26 (1.00–1.62)	<0.0001
eGFR CKD-EPI (mL/min/1.73 m^2^)	59 (42–82)	79 (60–92)	51 (37–70)	<0.0001
eGFR CKD-EPI < 60 mL/min/1.73 m^2^	182 (51.1)	27 (24.1)	155 (63.5)	<0.0001
**Diagnosis:**				
Cardiac non-coronary	272 (76.4)	84 (75.0)	188 (77.0)	NS
Non-cardiac	84 (23.6)	28 (25.0)	56 (23.0)	NS

ACS, acute coronary syndrome; AMI, acute myocardial infarction; AP, angina pectoris; CKD-EPI, Chronic Kidney Disease Epidemiology Collaboration; eGFR, estimated glomerular filtration rate; EP, endpoint; GRACE, Global Registry of Acute Coronary Events; hs-cTnT, high-sensitivity cardiac troponin T; NS, non significant; (N)STEMI, (non-)ST-elevation myocardial infarction. All continuous variables are given as median (interquartile range). * comparison of copeptin ≤ 10 pmol/L vs. >10 pmol/L.

**Table 2:** Outcomes.

**All Patients**	**All Patients** **(*n* = 1956)**	**Copeptin** **≤10 pmol/L** **(*n* = 782)**	**Copeptin** **>10 pmol/L** **(*n* = 1174)**	*p*-Value *
**Outcome**				
Death	425 (21.7)	84 (10.7)	341 (29.0)	<0.0001
Death 30 days	62 (3.2)	4 (0.5)	58 (4.9)	<0.0001
Death 90 days	112 (5.7)	12 (1.5)	100 (8.5)	<0.0001
Death 365 days	211 (10.8)	35 (4.5)	176 (15.0)	<0.0001
Cardiac death	118 (6.0)	18 (2.3)	100 (8.5)	<0.0001
MI	241 (12.3)	74 (9.5)	167 (14.2)	0.0033
Death/MI/stroke	641 (32.8)	161 (20.6)	480 (40.9)	<0.0001
Cardiac death/MI	330 (16.9)	87 (11.1)	243 (20.7)	<0.0001
Death/Resuscitation	446 (22.8)	92 (11.8)	354 (30.2)	<0.0001
**ACS**	**All patients** **(*n* = 1600)**	**Copeptin** **≤10 pmol/L** **(*n* = 670)**	**Copeptin** **>10 pmol/L** **(*n* = 930)**	*p*-Value *
**Outcome**				
Death	328 (20.5)	71 (10.6)	257 (27.6)	<0.0001
Death 30 days	45 (2.8)	3 (0.4)	42 (4.5)	<0.0001
Death 90 days	83 (5.2)	9 (1.3)	74 (8.0)	<0.0001
Death 365 days	151 (9.4)	26 (3.9)	125 (13.4)	<0.0001
Cardiac death	92 (5.7)	16 (2.4)	76 (8.2)	<0.0001
MI	218 (13.6)	72 (10.7)	146 (15.7)	0.0081
Death/MI/stroke	525 (32.8)	145 (21.6)	380 (40.9)	<0.0001
Cardiac death/MI	284 (17.8)	83 (12.4)	201 (21.6)	<0.0001
Death/Resuscitation	346 (21.6)	78 (11.6)	268 (28.8)	<0.0001
**Non-ACS**	**All patients** **(*n* = 356)**	**Copeptin** **≤10 pmol/L** **(*n* = 112)**	**Copeptin** **>10 pmol/L** **(*n* = 244)**	*p*-Value *
**Outcome**				
Death	97 (27.2)	13 (11.6)	84 (34.4)	0.0001
Death 30 days	17 (4.8)	1 (0.9)	16 (6.6)	0.0231
Death 90 days	29 (8.1)	3 (2.7)	26 (10.7)	0.0143
Death 365 days	60 (16.9)	9 (8.0)	51 (20.9)	0.006
Cardiac death	26 (7.3)	2 (1.8)	24 (9.8)	0.0091
MI	23 (6.5)	2 (1.8)	21 (8.6)	0.0187
Death/MI/stroke	116 (32.6)	16 (14.3)	100 (41.0)	<0.0001
Cardiac death/MI	46 (12.9)	4 (3.6)	42 (17.2)	0.0009
Death/Resuscitation	100 (28.1)	14 (12.5)	86 (35.2)	0.0002

MI, myocardial infarction; *, comparison of copeptin ≤10 pmol/L vs. >10 pmol/L.

**Table 3:** Cox proportional hazards model for all-cause mortality.

**(A) Entire Cohort.**			
**Univariate**	**HR**	**95% CI**	***p*-Value**
Age ≥ 70 years	3.85	3.05–4.85	<0.0001
eGFR < 60 mL/min	3.98	3.27–4.85	<0.0001
GRACE-Score ≥ 140	4.51	3.72–5.48	<0.0001
AMI diagnosis	0.74	0.61–0.9	0.004
Symptom onset < 3 h	0.78	0.60–1.02	NS
Symptom dyspnea	2.05	1.68–2.49	<0.0001
Hs-TnT 0h tertiles	1.19	1.06–1.34	0.0041
Copeptin > 10 pmol/L	2.99	2.36–3.80	<0.0001
**Multivariate**	**HR**	**95% CI**	***p*-Value**
Age ≥ 70 years	2.15	1.65–2.79	<0.0001
eGFR < 60 mL/min	1.78	1.42–2.25	<0.0001
GRACE-Score ≥ 140	2.13	1.71–2.66	<0.0001
AMI diagnosis	0.75	0.59–0.97	0.025
Symptom onset < 3 h	1.07	0.97–1.17	NS
Symptom dyspnea	1.37	1.11–1.69	0.003
Hs-TnT 0 h tertiles	1.46	1.26–1.68	<0.0001
Copeptin > 10 pmol/L	1.97	1.55–2.57	<0.0001
**(B) ACS Cohort.**			
**Univariate**	**HR**	**95% CI**	***p*-Value**
Age ≥ 70 years	4.46	3.41–5.84	<0.0001
eGFR < 60 mL/min	4.59	3.67–5.73	<0.0001
GRACE-Score ≥ 140	4.87	3.90–6.08	<0.0001
STEMI diagnosis	0.75	0.57–0.99	0.04
Symptom onset < 3 h	0.90	0.67–1.19	NS
Symptom dyspnea	1.92	1.54–2.38	<0.0001
Hs-TnT 0 h tertiles	1.30	1.13–1.49	0.0002
Copeptin > 10 pmol/L	2.82	2.17–3.66	<0.0001
**Multivariate**	**HR**	**95% CI**	***p*-Value**
Age ≥ 70 years	2.49	1.84–3.38	<0.0001
eGFR < 60 mL/min	2.18	1.68–2.83	<0.0001
GRACE-Score ≥ 140	2.02	1.57–2.60	<0.0001
STEMI diagnosis	0.92	0.68–1.24	NS
Symptom onset < 3 h	1.00	0.90–1.11	NS
Symptom dyspnea	1.31	1.05–1.65	0.0188
Hs-TnT 0 h tertiles	1.43	1.24–1.64	<0.0001
Copeptin > 10 pmol/L	1.72	1.30–2.29	0.0002
**(C) Non-ACS Cohort.**			
**Univariate**	**HR**	**95% CI**	***p*-Value**
Age ≥ 70 years	1.98	1.25–3.13	0.003
eGFR < 60 mL/min	1.80	1.19–2.73	0.006
GRACE-Score ≥ 140	3.01	2.02–4.49	<0.0001
Symptom onset < 3 h	0.41	0.15–1.11	NS
Symptom dyspnea	1.76	1.00–3.11	NS
Hs-TnT 0 h tertiles	1.55	1.20–2.01	0.0009
Copeptin > 10 pmol/L	3.24	1.80–5.81	0.0001
**Multivariate**	**HR**	**95% CI**	***p*-Value**
Age ≥ 70 years	1.38	0.80–2.37	NS
eGFR < 60 mL/min	0.82	0.51–1.33	NS
GRACE-Score ≥ 140	2.09	1.30–3.35	0.0022
Symptom onset < 3 h	1.30	1.01–1.68	0.0405
Symptom dyspnea	1.19	0.66–2.15	NS
Hs-TnT 0 h tertiles	1.53	1.16–2.03	0.003
Copeptin > 10 pmol/L	2.69	1.45–5.02	0.0018

AMI, acute myocardial infarction; CI, confidence interval; eGFR, estimated glomerular filtration rate; GRACE, Global Registry of Acute Coronary Events; hs-cTnT, high-sensitivity cardiac troponin T, HR, hazard-ratio; NS, non-significant.

## Supplementary Materials

The following are available online at https://www.mdpi.com/2077-0383/9/11/3627/s1, Figure S1: Study flow-chart; Table S1: Categories of adjudicated non-ACS diagnoses; Table S2: Cox proportional hazard models for the combined endpoint of death, myocardial infarction, and stroke; Table S3: Prognostic measures of copeptin for prediction of all-cause death (A) and the combined endpoint of death/MI/stroke (B); Table S4: Prognostic performance of hs-cTnT (A) and GRACE risk score (B) with and without addition of copeptin.
