# Supplementary material for: Prognostic Value of Elevated Copeptin and High-Sensitivity Cardiac Troponin T in Patients with and without Acute Coronary Syndrome: The ConTrACS Study
Source: J Clin Med. 2020 Nov 11;9(11):3627. doi: 10.3390/jcm9113627 (PMC7696893; doi:10.3390/jcm9113627)
Supplement: Supplementary file 1 [file jcm-09-03627-s001.pdf]

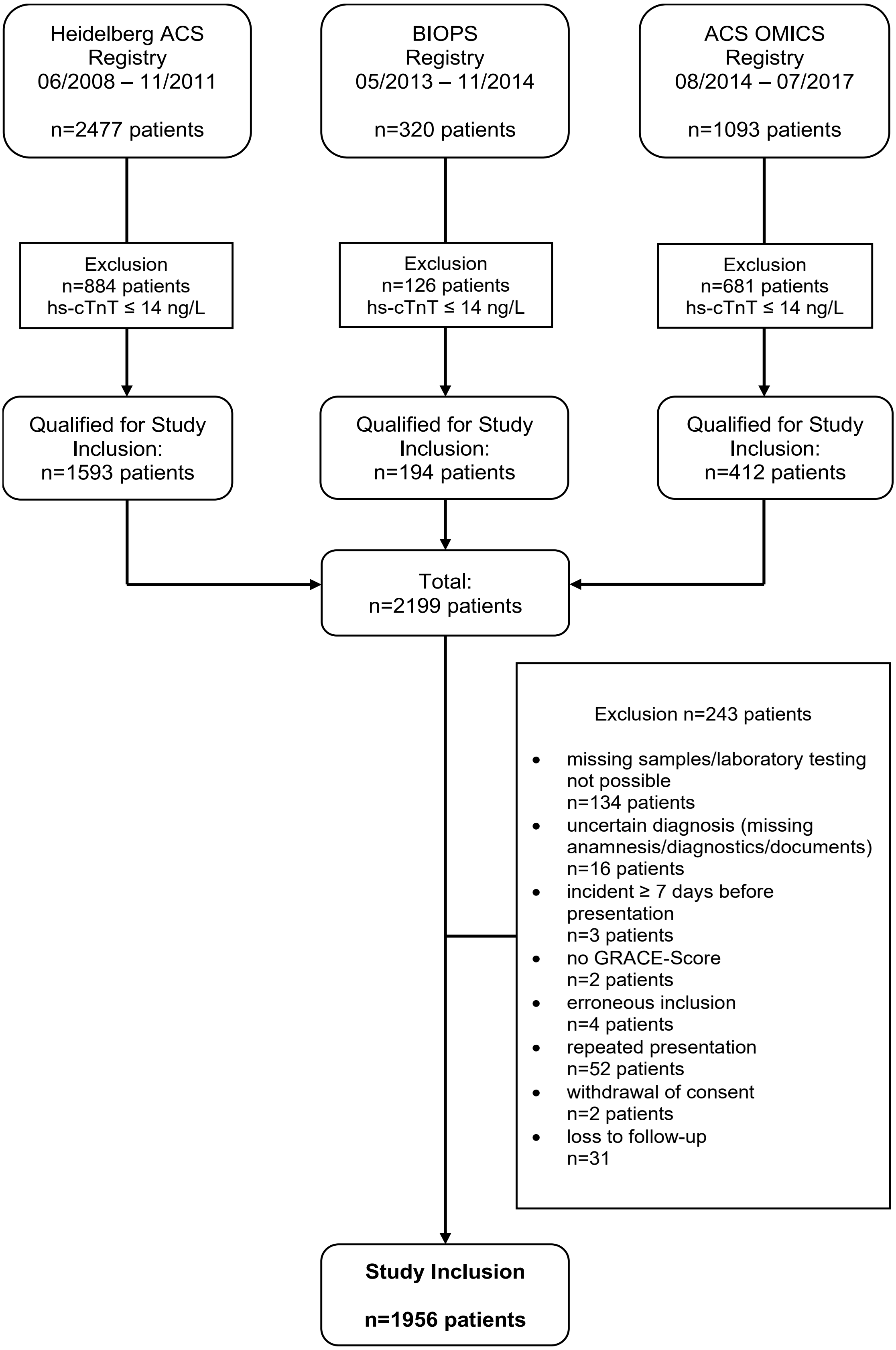

Supplementary Materials Figure S1. Study flow-chart.

Detailed overview on the initial study cohorts:

ACS Registry and ACS OMICS Registry
Full Titles:
Cardiac biochemical and genetic markers in patients with acute coronary syndrome in the emergency department (Heidelberg ACS Registry)
Cardiac biochemical and omics-based markers in patients with symptoms suggestive of acute coronary syndrome in the emergency department (ACS OMICS Registry)
Objective:
The Heidelberg ACS Registry was initiated to differentiate acute myocardial infarction (AMI) from non-AMI-related diagnoses and assess the prognostic outcome based on novel cardiovascular biomarkers. The ACS OMICS Registry is the continuation of the Heidelberg ACS Registry. Both registries therefore followed the same study protocol.
Participating Centers: Heidelberg University Hospital
Study Population:
Patients presenting with symptoms suggestive of acute coronary syndrome to the emergency department
Study Type: single-center, prospective, observational
Ethics committee approvals: S-3302003 and S-3512015 (University of Heidelberg)
Primary Outcome Measures:
Diagnostic utility of established and novel cardiovascular biomarkers for detection of myocardial infarction
Secondary Outcome Measures:
Prognostic performance of established and novel cardiovascular biomarkers for prediction of adverse outcomes (all-cause death, cardiovascular death, non-fatal myocardial infarction, non-fatal stroke)
Study Protocol:
Patients receive assessment of history, physical examination, a comprehensive laboratory testing (including high-sensitivity cardiac troponin T), and electrocardiogram (ECG) as part of clinical routine. Additional work-up, such as chest X-ray, computed-tomography (CT) scan, echocardiography, or invasive cardiac procedures, are left at the discretion of the attending emergency physician. Study participation also includes blood sampling at least at the time of presentation for measurement of additional biomarkers. Gold standard diagnosis is adjudicated by two trained cardiologists using all available clinical and imaging parameters (e.g., laboratory testing, ECG, Echo, coronary angiography, etc.). A third cardiologist referred in cases of disagreement. All patients are contacted by telephone or written contact at 30 days and 1 year after discharge. Additional follow-up is obtained by review of the hospital information system and/or by requesting information of survival at the local residents' registration offices.
Sampling specimen: Plasma, serum, citrate
General Inclusion Criteria:

- Acute symptoms suggestive of acute coronary syndrome
- Age≥18 years

General Exclusion Criteria:

- Age <18 years
- Inability to give informed consent
- Cardiogenic shock or other diseases requiring immediate medical treatment
- Pregnancy/lactation period

Specific-Inclusion Criteria (for ConTrACS analysis):

- Available hs-cTnT and copeptin values at presentation or available blood samples at presentation for re-measurement

Specific Exclusion Criteria (for ConTrACS analysis):

- Absence of available hs-cTnT and copeptin values at presentation or inability for re-measurement due to missing blood samples

- Missing clinical data for determination of adjudicated diagnosis and/or GRACE score calculation
- Repeated presentation
- Loss of follow-up <30 days
- Delayed presentation (>7 days after incident)

BIOPS-Registry
Full Title: Biomarkers in patients presenting with shortness of breath (BIOPS)
Objective:
To assess the diagnostic and prognostic performance of cardiovascular biomarkers to differentiate acute heart failure from other cardiac or non-cardiac diseases in the emergency department.
Participating Centers: Heidelberg University Hospital
Study Population:
Patients presenting to the emergency department with new onset of dyspnea or worsening of chronic dyspnea within the last 2 weeks
Study Type: single-center, prospective, observational
Ethics committee approval: S-1172013 (University of Heidelberg)
Primary Outcome Measures:
Diagnostic utility of established and novel cardiovascular biomarkers to diagnose acute heart failure.
Secondary Outcome Measures:
Prognostic performance of established and novel cardiovascular biomarkers for prediction of adverse outcomes (all-cause death, cardiovascular death, non-fatal myocardial infarction, non-fatal stroke)
Study Protocol:
Patients receive assessment of history, physical examination, a comprehensive laboratory testing (including high-sensitivity cardiac troponin T), and ECG as part of clinical routine. Additional work-up, such as chest X-ray, CT scan, echocardiography, or invasive cardiac procedures, is left at the discretion of the attending emergency physician. Study participation also includes blood sampling at least at the time of presentation for measurement of additional biomarkers. Gold standard diagnosis is adjudicated by two trained cardiologists using all available clinical and imaging parameters (e.g., laboratory testing, ECG, Echo, coronary angiography, etc.). A third cardiologist is referred in cases of disagreement. All patients are contacted by telephone or written contact at 30 days and 90 days after discharge. Additional follow-up is obtained by review of the hospital information system and/or by requesting information of survival at the local residents' registration offices
Sampling specimen: Plasma, serum, citrate
General Inclusion Criteria:
- New onset of dyspnea or worsening of chronic dyspnea within the last 2 weeks
- Age≥18 years

General Exclusion Criteria:
- Age <18 years
- Inability to give informed consent
- Cardiogenic shock or other diseases requiring immediate medical treatment
- Pregnancy/lactation period

Specific-Inclusion Criteria (for ConTrACS analysis):
- Available hs-cTnT and copeptin values at presentation or available blood samples at presentation for re-measurement

Specific Exclusion Criteria (for ConTrACS analysis):
- Absence of available hs-cTnT and copeptin values at presentation or inability for re-measurement due to missing blood samples
- Missing clinical data for determination of adjudicated diagnosis and/or GRACE score calculation
- Repeated presentation
- Loss of follow-up <30 days

- Delayed presentation (>7 days after incident)

Supplementary Materials Table S1. Categories of adjudicated non-ACS diagnoses.

Diagnostic categories of non-ACS patients (*n*=356)	Frequency
Brady-/Tachyarrhythmia	29 (8.1%)
Valvular heart disease	37 (10.4%)
Heart failure	139 (39.0%)
Cardiomyopathies (including tako tsubo)	33 (9.3%)
Myo-/Peri-/Endocarditis	16 (4.5%)
Hypertensive crisis	9 (2.5%)
Pulmonary embolism	30 (8.4%)
Pneumonia/AECOPD	34 (9.6%)
Miscellaneous	29 (8.2%)

AECOPD, acute exacerbated chronic obstructive pulmonary disease; all numbers are given as absolute frequency (relative frequency).

Supplementary Materials Table S2. Cox proportional hazard models for the combined endpoint of death, myocardial infarction, and stroke.

A) entire cohort

Univariate	HR	95% CI	p-value
Age≥ 70 years	2.47	2.08–2.94	< 0.0001
eGFR < 60 ml/min	2.89	2.47–3.38	< 0.0001
GRACE-Score ≥ 140	3.07	2.61–3.61	< 0.0001
AMI diagnosis	0.89	0.75–1.05	NS
Symptom onset<3h	0.85	0.69–1.05	NS
Symptom dyspnea	1.78	1.52–2.08	< 0.0001
Hs-TnT 0h tertiles	1.14	1.03–1.26	0.0082
Copeptin > 10 pmol/L	2.17	1.81–2.59	< 0.0001

Multivariate	HR	95% CI	p-value
Age≥ 70 years	1.58	1.29–1.92	< 0.0001
eGFR < 60 ml/min	1.67	1.38–2.02	< 0.0001
GRACE-Score ≥ 140	1.77	1.47–2.13	< 0.0001
AMI diagnosis	0.95	0.77–1.16	NS
Symptom onset<3h	1.04	0.97–1.12	NS
Symptom dyspnea	1.34	1.13–1.58	0.0007
Hs-TnT 0h tertiles	1.27	1.13–1.42	0.0001
Copeptin > 10 pmol/L	1.58	1.30–1.91	< 0.0001

B) ACS cohort

Univariate	HR	95% CI	p-value
Age≥ 70 years	2.59	2.14–3.13	< 0.0001
eGFR < 60 ml/min	3.23	2.72–3.84	< 0.0001
GRACE-Score ≥ 140	3.12	2.61–3.74	< 0.0001
STEMI diagnosis	0.64	0.51–0.80	0.0001
Symptom onset<3h	0.89	0.71–1.11	NS
Symptom dyspnea	1.77	1.49–2.10	< 0.0001

Hs-TnT 0h tertiles	1.13	1.02–1.26	0.0233
Copeptin > 10 pmol/L	2.03	1.68–2.46	< 0.0001

**Multivariate**	**HR**	**95% CI**	**p-value**
Age≥ 70 years	1.57	1.26–1.95	0.0001
eGFR < 60 ml/min	1.89	1.54–2.32	< 0.0001
GRACE-Score ≥ 140	1.69	1.37–2.07	< 0.0001
STEMI diagnosis	0.75	0.59–0.96	0.021
Symptom onset<3h	1.00	0.92–1.08	NS
Symptom dyspnea	1.30	1.09–1.56	0.0043
Hs-TnT 0h tertiles	1.21	1.09–1.35	0.0005
Copeptin > 10 pmol/L	1.50	1.22–1.85	0.0001

C) non-ACS cohort

**Univariate**	**HR**	**95% CI**	**p-value**
Age≥ 70 years	1.85	1.22–2.80	0.004
eGFR < 60 ml/min	1.64	1.12–2.40	0.01
GRACE-Score ≥ 140	2.73	1.89–3.95	< 0.0001
Symptom onset<3h	0.67	0.32–1.37	NS
Symptom dyspnea	1.73	1.04–2.87	0.034
Hs-TnT 0h tertiles	1.49	1.17–1.88	0.0011
Copeptin > 10 pmol/L	3.05	1.80–5.18	< 0.0001
**Multivariate**	**HR**	**95% CI**	**p-value**
Age≥ 70 years	1.37	0.84–2.24	NS
eGFR < 60 ml/min	0.78	0.50–1.20	NS
GRACE-Score ≥ 140	1.99	1.29–3.07	0.0019
Symptom onset<3h	1.19	0.96–1.48	NS
Symptom dyspnea	1.25	0.73–2.12	NS
Hs-TnT 0h tertiles	1.48	1.14–1.91	0.003
Copeptin > 10 pmol/L	2.61	1.49–4.58	0.0008

AMI, acute myocardial infarction; CI, confidence interval; eGFR, estimated glomerular filtration rate; GRACE, Global Registry of Acute Coronary Events; hs-cTnT, high-sensitivity cardiac troponin T

Supplementary Materials Table S3. Prognostic measures of copeptin for prediction of all-cause death (A) and the combined endpoint of death/MI/stroke (B).

A)

	**All-cause death**				
	**Sensitivity# [%]**	**Specificity# [%]**	**PPV# [%]**	**NPV# [%]**	**AUC**
Entire cohort	80.2 (76.1–83.9)	45.6 (43.1–48.1)	29.0 (27.7–30.4)	89.3 (87.2–91.0)	0.68* (0.66–0.70)
ACS	78.4 (73.5–82.7)	47.1 (44.3–49.9)	27.6 (26.1–29.2)	89.4 (87.2–91.3)	0.68* (0.66–0.70)
Non-ACS	86.6 (78.2–92.7)	37.8 (31.9–44.9)	34.3 (31.6–37.1)	88.3 (81.6–92.8)	0.68* (0.63–0.73)

B)

B)	Death/MI/stroke				
	**Sensitivity# [%]**	**Specificity# [%]**	**PPV# [%]**	**NPV# [%]**	**AUC**
Entire cohort	74.9 (71.3–78.2)	47.2 (44.5–50.0)	40.9 (39.3–42.5)	79.4 (76.9–81.7)	0.64* (0.62–0.67)
ACS	72.4 (68.3–76.2)	48.8 (45.8–51.9)	40.9 (39.0–42.8)	78.4 (75.7–80.8)	0.64* (0.61–0.66)
Non-ACS	86.2 (78.6–91.9)	39.6 (33.4–46.1)	40.8 (37.8–43.9)	85.6 (78.6–90.6)	0.68* (0.63–0.73)

ACS; acute coronary syndrome; AUC, area-under-the-curve; MI, myocardial infarction; NPV, negative predictive value; PPV, positive predictive value. # cut-off >10 pmol/L; *p-value<0.0001

Supplementary Materials Table S4. Prognostic performance of hs-cTnT (A) and GRACE risk score (B) with and without addition of copeptin.

A)

**Death**	**AUC**		**p-value**
	**Hs-cTnT**	**Hs-cTnT & Copeptin**	
Entire cohort	0.56 (0.56–0.61)	0.68 (0.66–0.70)	<0.0001
ACS	0.60 (0.58–0.62)	0.68 (0.65–0.70)	0.0001
Non–ACS	0.63 (0.58–0.68)	0.68 (0.63–0.73)	NS
**Death/MI/stroke**	**AUC**		**p-value**
	**Hs-cTnT**	**Hs-cTnT & Copeptin**	
Entire cohort	0.57 (0.54–0.59)	0.64 (0.62–0.66)	<0.0001
ACS	0.56 (0.53–0.58)	0.63 (0.61–0.66)	<0.0001
Non-ACS	0.63 (0.58–0.68)	0.68 (0.63–0.73)	NS

B)

**Death**	**AUC**		**p-value**
	**GRACE**	**GRACE & Copeptin**	
Entire cohort	0.76 (0.74–0.78)	0.77 (0.75–0.79)	0.0087
ACS	0.77 (0.75–0.79)	0.78 (0.76–0.80)	0.04
Non-ACS	0.69 (0.64–0.74)	0.70 (0.65–0.74)	NS
**Death/MI/stroke**	**AUC**		**p-value**
	**GRACE**	**GRACE & Copeptin**	
Entire cohort	0.71 (0.69–0.73)	0.72 (0.70–0.74)	NS
ACS	0.73 (0.70–0.75)	0.73 (0.70–0.75)	NS
Non-ACS	0.67 (0.62–0.72)	0.68 (0.62–0.72)	NS

ACS; acute coronary syndrome; AUC, area-under-the-curve; GRACE, Global Registry of Acute Coronary Events; hs-cTnT, high-sensitivity cardiac troponin T; MI, myocardial infarction
